# Analysis and occurrence of biallelic pathogenic repeat expansions in *RFC1* in a German cohort of patients with a main clinical phenotype of motor neuron disease

**DOI:** 10.1007/s00415-024-12519-6

**Published:** 2024-06-25

**Authors:** Annalisa Schaub, Hannes Erdmann, Veronika Scholz, Manuela Timmer, Isabell Cordts, Rene Günther, Peter Reilich, Angela Abicht, Florian Schöberl

**Affiliations:** 1 Medical Genetics Center, Munich, Germany; 2 Department of Neurology With Friedrich-Baur-Institute, Klinikum Der Universität, Ludwig-Maximilians-University, Marchioninistr. 15, 81377 Munich, Germany; 3 Gemeinschaftspraxis Für Humangenetik Dresden, Medizinische Genetik, Dresden, Germany; 4 Department of Neurology, Klinikum Rechts Der Isar, Technical University of Munich, Munich, Germany; 5 Department of Neuroscience, Mayo Clinic, Jacksonville, FL USA; 6 Department of Neurology, Universitätsklinikum Carl Gustav Carus Dresden, Dresden, Germany

**Keywords:** Repeat analysis, Oxford nanopore sequencing, Amyotrophic lateral sclerosis, RFC1, MND, Motor neuron disease

## Abstract

Biallelic pathogenic repeat expansions in *RFC1* were recently identified as molecular origin of cerebellar ataxia, neuropathy, vestibular areflexia syndrome (CANVAS) as well as of one of the most common causes of adult-onset ataxia. In the meantime, the phenotypic spectrum has expanded massively and now includes mimics of multiple system atrophy or parkinsonism. After identifying a patient with a clinical diagnosis of amyotrophic lateral sclerosis (ALS) as a carrier of biallelic pathogenic repeat expansions in *RFC1*, we studied a cohort of 106 additional patients with a clinical main phenotype of motor neuron disease (MND) to analyze whether such repeat expansions are more common in MND patients. Indeed, two additional MND patients (one also with ALS and one with primary lateral sclerosis/PLS) have been identified as carrier of biallelic pathogenic repeat expansions in *RFC1* in the absence of another genetic alteration explaining the phenotype, suggesting motor neuron disease as another extreme phenotype of *RFC1* spectrum disorder. Therefore, MND might belong to the expanding phenotypic spectrum of pathogenic *RFC1* repeat expansions, particularly in those MND patients with additional features such as sensory and/or autonomic neuropathy, vestibular deficits, or cerebellar signs. By systematically analyzing the *RFC1* repeat array using Oxford nanopore technology long-read sequencing, our study highlights the high intra- and interallelic heterogeneity of this locus and allows the identification of the novel repeat motif ‘ACAAG’.

**Supplementary Information:**

The online version contains supplementary material available at 10.1007/s00415-024-12519-6.

## Introduction

In 2019, biallelic pathogenic repeat expansions in intron 2 of the *replication factor C subunit 1* gene (*RFC1*) have been identified as a common genetic cause of cerebellar ataxia, neuropathy, vestibular areflexia syndrome (CANVAS) and were shown to be a major cause of hereditary late-onset ataxia [1, 2]. Unlike other repeat expansion disorders, *RFC1* spectrum disorder does not only require the expansion of a wild-type motif but also its substitution for a pathogenic motif (most commonly ‘AAGGG’). The *RFC1* locus is highly polymorphic. To date, at least 11 motifs are known with 6 of which are likely pathogenic when largely expanded [3–7].

The phenotypic spectrum associated with these repeat expansions is broad and includes chronic cough, sensory neuropathy, cerebellar ataxia, bilateral vestibular failure, and autonomic dysfunctions, among others [8, 9]. Interestingly, biallelic repeat expansions in *RFC1* have also been associated with several other distinct neurodegenerative disorders, such as Parkinson’s disease and atypical parkinsonism such as multiple system atrophy (MSA) [10–12]. Recently, the clinical phenotypic spectrum of *RFC1*-related disorders has been further extended by the report of upper and lower motor neuron involvement observed clinically as well as in post-mortem neuropathological examinations [13, 14]. Furthermore, a case of ALS and additional sensory neuropathy and presbyvestibulopathy in the presence of biallelic pathogenic *RFC1* motifs has been reported [15].

This raises the question of whether *RFC1* should be added to the list of MND-related genes and considered in the genetic diagnostic process. This is particularly relevant, considering that the etiology of sporadic MND such as amyotrophic lateral sclerosis (ALS) and primary lateral sclerosis (PLS) remains unexplained in up to ~ 90% of cases, and biallelic repeat expansions in *RFC1*, an autosomal-recessive disease, may potentially offer an additional genetic explanation in at least a subset of those patients [16–18].

Therefore, in this study, we conducted a systematic screening for *RFC1* expansions in a cohort of 107 patients with MND primarily composed of ALS patients employing CRISPR/Cas9-targeted Oxford nanopore technology (ONT) long-read sequencing [4, 19]. This method allowed not only an in-depth analysis of *RFC1*, evaluating repeat motifs and accurate repeat lengths in a large cohort, but also a combined assessment of the important ALS-related *C9orf72* repeat array. In addition, we applied a comprehensive high-throughput sequencing gene panel of 380 MND-related genes to all individuals to exclude additional relevant and so far known underlying genetic alterations.

## Materials and methods

### Patient cohort

All patients received diagnostic testing as ordered by their attending physicians (e.g., neurologist, geneticist). Informed consent was signed and obtained in all the cases (see ethical standards). Patients were informed about the findings and their significance for relatives. Genetic counseling was recommended and offered in all these cases.

### Extraction of genomic DNA (gDNA)

Genomic DNA (gDNA) of 107 patients with a clinical phenotype of MND was obtained from total peripheral EDTA blood samples by extraction of white blood cells with a Biomek FX system (Beckman Coulter) using the NucleoMag® Blood 3 ml Kit (Machery-Nagel, #REF 744502.1) as per manufacturer’s instructions. All DNA samples showed high purity as determined by optical density measurements (A260/A280 > 1.9 and A260/A230 > 2.0). The diagnosis of MND was made exclusively by experienced neurologists in outpatient clinics specialized in motor neuron disease or neuromuscular diseases. The corresponding diagnoses were as follows: ALS according to Gold Coast Criteria (*n* = 76; 71.0%), PLS according to Turner et al. (*n* = 16; 15.0%), progressive muscular atrophy, PMA (*n* = 7; 6.5%), MND (*n* = 8; 7.5%) (see Suppl. Figure 1) [20, 21]. PMA was diagnosed after thorough exclusion of alternative diagnosis (i.e., polyneuropathy, SBMA, adult-onset SMA, dHMN, and myopathy).

### Repeat analysis of RFC1, C9orf72, and AR by Oxford nanopore technology long-read sequencing

All patients were analyzed by an Oxford nanopore technology long-read sequencing-based repeat analysis to identify pathogenic repeat expansions in *C9orf72*, *RFC1* and *AR* for Kennedy’s disease. Library preparation and flow cell loading were performed according to the Oxford nanopore technology (ONT) Cas9-targeted sequencing protocol using ONT’s SQK-CS9109 kit and 5 µg of input gDNA. CRISPR RNAs (crRNAs) to enrich *C9orf72*, *RFC1*, and *AR* were designed using CHOPCHOP 8 (Supplementary Table 1). Sequencing was performed with ONT FLO-MIN106D R9 flow cells on the GridION X5 sequencer.

Data were analyzed as previously described [4]. Base calling from electrical data was performed using Guppy (v5.0.16) [22, 23]. The generated FASTQ files were aligned to the human reference genome (GRCh38/hg38) using Minimap2 (v2.17) to identify the reads spanning the targets of interest [24]. For quality control of the aligned reads, we used NanoPlot (v1.29.1) [25]. The bioinformatics tool STRique (v0.2.1) was used to determine the number of repeat units (RU) for all reads assigned to *C9orf72* [26]. Repeat size distributions obtained by STRique were visualized as violin plots and used to determine the repeat size for each allele by computing local maxima using FindPeaks (v2.1.1) and visual assessment of the plots [27]. For extended repeat expansion (> 100 RU) showing multiple local maxima, a size range was calculated starting with the lowest and ending with the highest significant peak as determined by FindPeaks. Only fragments spanning the entire repeat were considered for the repeat length quantification. The *RFC1* locus was analyzed manually by visual inspection of all reads mapped to that region in the IGV. Repeat lengths distribution as well as average repeat sizes were extracted for each allele separately. Patients being heterozygous for a pathogenic repeat expansion in *RFC1* were analyzed for a pathogenic variant in *trans* by NGS sequencing as rare cause of *RFC1* spectrum disorder.

### High-throughput sequencing and bioinformatics pipeline

To identify pathogenic single nucleotide variants (SNVs) or copy number variations (CNVs) in known, altogether 380 MND-related genes, gene targeted enrichment was performed with the SureSelectXT gene panel custom kit (Agilent Biosciences) or the Twist Human Comprehensive Exome Kit (Twist Biosciences). Massively parallel sequencing was carried out on an Illumina NextSeq 500 or a Novaseq 6000 system (Illumina, San Diego, CA) as 150 bp paired-end runs using v2.0 SBS chemistry. Secondary and tertiary analysis was carried out using varvis® 1.22.0. Pipeline versions fc9-063-00b and 8a2-0c8-080 were used for SNV and CNV analysis, respectively. The 380 MND-related genes analyzed are summarized in Table 2 of the Supplement. In addition, *RFC1* was analyzed in all patients with a heterozygous pathogenic repeat expansion in *RFC1*. Homozygous deletion in *SMN1* was excluded by a masking pipeline as previously described [28].

Only SNVs and small insert and deletions (INDELs) in the coding and flanking intronic regions (± 50 bp) were evaluated. Variants were classified according to the ACMG (American College of Medical Genetics and genomics) guidelines [29]. Likely pathogenic and pathogenic variants are summarized as P/LP variants.

### Data availability

Anonymized data from this study are available from the corresponding author on reasonable request.

## Results

### Identification of known MND-related genetic alterations

**Fig. 1:**
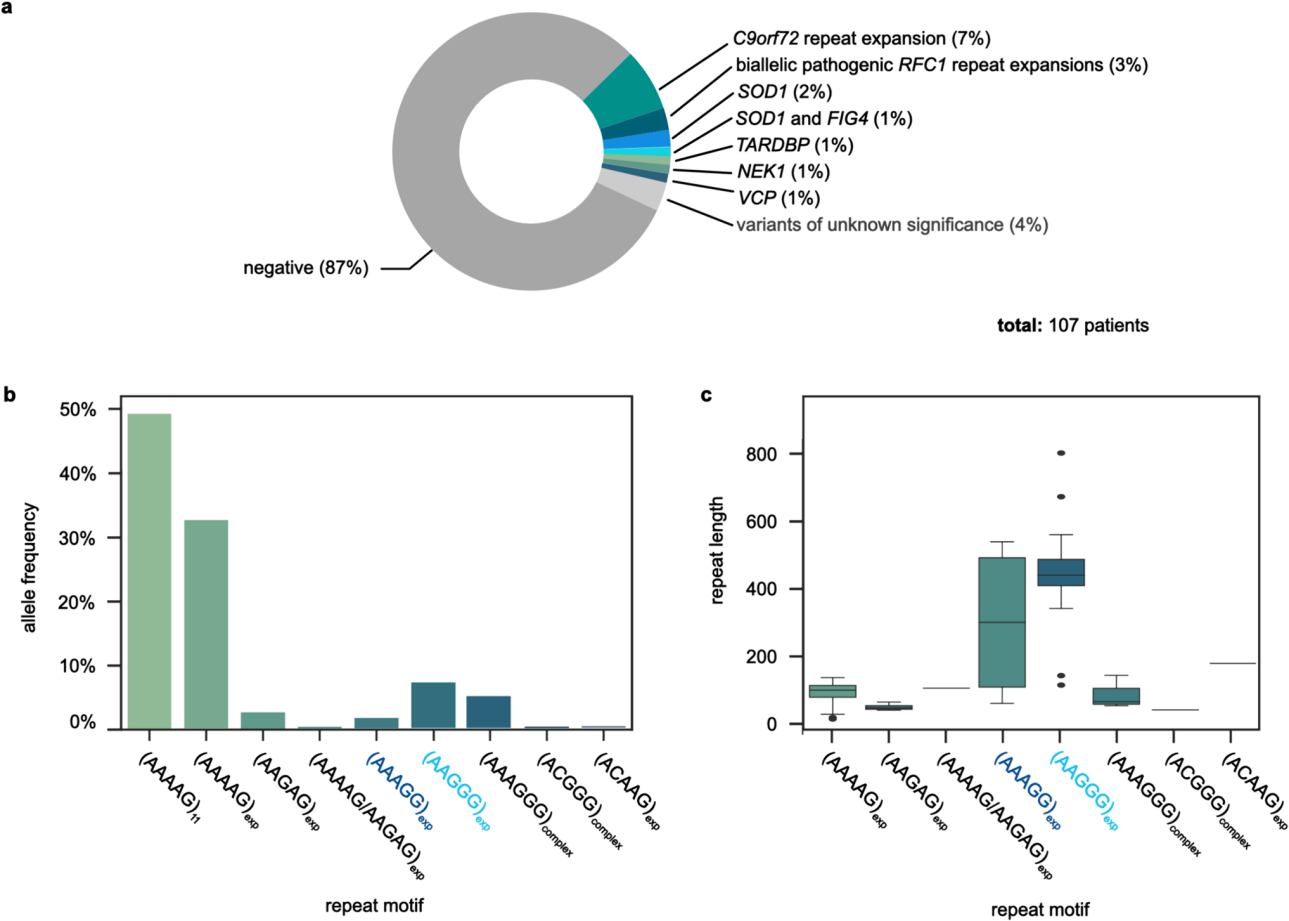
Results of the genetic analysis of 107 patients with MND. (**a**) Genetic causes of MND identified by long-read sequencing-based repeat analysis and NGS sequencing of MND-related genes. (**b**) Repeat motifs of the *RFC1* repeat array identified in all patients analyzed and their frequency. Pathogenic motif in light blue. Motif with size-dependent pathogenicity in dark blue. (**c**) Repeat length distribution of the different repeat motifs of the *RFC1* locus determined in the cohort studied

To identify known genetic alterations associated with MND, all 107 patients underwent parallel repeat analysis by ONT long-read sequencing of the ALS-related *C9orf72* repeat array and the *AR* locus associated with spinal and bulbar muscular atrophy (SBMA/Kennedy’s disease) as well as NGS sequencing of MND-related genes (Fig. 1, Supplementary Table 3) [20]. Overall, in 14 individuals (13%), a related genetic alteration could be identified. In 8/107 patients (7.5%), a pathogenic repeat expansion in *C9orf72* was detected [30]. None of the patients carried a pathogenic repeat expansion in *AR* associated with SBMA. Heterozygous P/LP variants in genes associated with ALS were identified in six patients: Three patients carried a variant in *SOD1*, with one patient additionally carrying a variant in *FIG4*. One patient each had a variant in *TARDBP*, in *VCP* or in *NEK1*, respectively. In addition, five patients had a variant of uncertain significance in ALS-related genes (*FUS*, *TBK1, SETX,* and *SOD1*; Supplementary Table 3). Neither P/LP nor variants of unclear significance (VUS) were found in any other of the altogether analyzed 380 genes associated with MND besides classical ALS with affection of only either the upper or lower motor neurons. Thus, in the majority of patients, no pathogenic or likely pathogenic variant or repeat expansion in a known ALS-related gene or genes associated with MND could be identified.

### In-depth analysis of the RFC1 repeat array in MND patients

*RFC1* repeat configurations (repeat motifs and repeat lengths) were determined for all patients (Fig. 1b, c, Supplementary Table 3) in parallel with *C9orf72* repeat analysis by ONT long-read sequencing. In three of the 107 MND patients (i.e., 2.8%), a biallelic pathogenic (AAGGG)_exp_ repeat expansion in *RFC1* was detected, one of which was previously reported by us [15]. No other genetic variants in any of the MND-related genes were detected in the panel analyses in these individuals. In all three individuals, the repeat length exceeded the currently established pathogenic cut-off of 250 repeat units [3]. Eight patients carried a pathogenic repeat configuration on one allele, but a non-pathogenic one on the other allele. In all these patients, pathogenic SNVs in *RFC1* in *trans* were excluded by NGS sequencing of *RFC1*.

We examined the frequency of the different repeat motifs (Fig. 1b) in *RFC1* and their repeat sizes (Fig. 1c) within the 214 alleles of our 107 patients. The unexpanded *RFC1* wild-type motif (AAAAG)_11_ was observed in 50% of the alleles. The expanded non-pathogenic wild-type motif (AAAAG)_exp_ was found in 32% of the alleles, ranging from 16 to 138 RU in size. The likely non-pathogenic motif (AAGAG)_exp_ was found in 2% of the alleles with a small variation in size from 42 to 66 RU. In addition, one polymorphic allele was found in which (AAGAG)_exp_ was surrounded by a stretch of (AAAAG)_exp_ at both sides. The motif with size-dependent pathogenicity (AAAGG)_exp_ was detected in 1% of the alleles with very different repeat lengths (126 RU, 476 RU and 539 RU). Considering the currently estimated threshold for pathogenicity of 600 RU for this motif, we classified all (AAAGG)_exp_ alleles detected as non-pathogenic [3]. The known pathogenic motif (AAGGG)_exp_ was detected in 7% of the alleles, ranging in size from 116 to 800 RU. Two of the alleles showed a size below the currently established threshold of pathogenicity of 250 RU [3].

In addition, complex polymorphic repeat configurations were identified in approximately 6% of the alleles. Intermittent sequences of (AAAGGG)_exp_ interrupted by (AAAGG)_exp_ and/or (AAGGG)_exp_ were detected in heterozygosity in 11 patients, all without a second pathogenic expansion on the other allele. One patient showed a complex (ACGGG)_exp_ repeat expansion interrupted by stretches of (AAAGG)_exp_ and (AAGGG)_exp_ in one allele, which has previously been reported as of unknown pathogenicity [3, 7]. However, this individual also carried a pathogenic repeat expansion in *C9orf72*. The two types of complex repeat expansions were annotated as (AAAGGG)_complex_ and (ACGGG)_complex_. In addition, we could identify the yet undescribed repeat configuration (ACAAG)_exp_ with a size of 180 RU heterozygous in one patient carrying the wild-type motif (AAAAG)_11_ on the other allele (Supplementary Table 3, Supplementary Fig. 2).

### Clinical presentation and diagnostic findings of MND patients with biallelic pathogenic repeat expansions in RFC1

The three male MND patients with a biallelic pathogenic repeat expansion in *RFC1* had a mean age of 59 years and a mean age of onset of 52 years. Two patients (patient #87 and #99) were diagnosed clinically with ALS fulfilling existing Gold Coast Criteria, while the other patient (patient #98) was diagnosed with definite PLS according to existing diagnostic criteria for PLS [20, 21]. The family history for MND was negative in all of them. In the two individuals with ALS, simultaneous and clear clinical signs of upper and lower motor neuron affection were detected including brisk and pathological tendon reflexes, as well as muscle weakness and atrophy with muscle fasciculations (Table 1). Both patients had a spinal onset of symptoms with one of them developing bulbar palsy with dysphagia, as well as dysarthria during disease progression. The third patient (patient #98) primarily presented with upper motor neuron affection manifesting with spasticity and pseudobulbar palsy fulfilling the diagnostic criteria for definite PLS [21]. This patient had no clinical signs of lower motor neuron affection (i.e., muscle atrophy and/or polytopic muscle fasciculations). By needle electromyography, there were only unspecific findings with single fasciculation potentials and mild to moderate chronic denervation in the anterior tibial muscle but no positive sharp waves and/or fibrillation potentials and no pathological findings in the examined muscles of the other body regions (bulbar–cervical–thoracic). Relevant differential diagnoses were excluded by magnetic resonance imaging of the brain and spinal cord (exclusion of lesions along the pyramidal tracts, brain stem and cervical/thoracic spinal cord), additional laboratory tests, exclusion of genetic alterations in MND-related genes, and particularly thorough electroneurography for exclusion of demyelinating immune neuropathy. Neurofilament light chain levels were analyzed in 2/3 patients either in serum or in cerebrospinal fluid, respectively, and found to be significantly elevated (see Table 1). Two patients showed autonomic dysfunction. After identification of biallelic pathogenic repeat expansions in *RFC1*, reverse phenotyping revealed additional symptoms of *RFC1* spectrum disorder in all patients. As such chronic cough (2/3 patients), sensory neuropathy (3/3 patients), cerebellar ataxia (1/3), as well as bilateral vestibulopathy (2/3) could be identified. While the progression of the disease in the patient previously described (patient #87) resembled that of a typical ALS course of disease, the two other patients showed a rather slow progression of motor symptoms: in one patient, they manifested more than a decade ago (patient #98) with an almost stable to slowly progressive course of disease with a late onset of chronic neurogenic changes as well as slight fasciculations and a similarly slow course of disease in the other patient for about 2 years (patient #99).

**Table 1:** Symptoms of three patients with *RFC1* spectrum disorder and a clinical main phenotype of MND

Patient	87	98	99
Gender	Male	Male	Male
Age	66	67	43
Age of onset	63	54	41
Affection of the upper motor neuron	Brisk muscle reflexes (cervical)	Brisk muscle reflexes (cervical, lumbosacral) Paraspasticity (lumbosacral) Pseudobulbar palsy (dysphagia and dysarthria)	Brisk muscle reflexes (cervical, lumbosacral)
Affection of the lower motor neuron	Muscle weakness and atrophy (cervical, thoracic, lumbosacral) Fasciculations (all 4 levels)	None	Muscle weakness and atrophy (cervical, lumbosacral) Fasciculations (lumbosacral) Bulbar palsy (dysphagia, dysarthria)
EMG	Acute denervation in all 4 levels	Chronic neurogenic changes Fasciculations in tibialis anterior muscle	No data available
Gold Coast Criteria (2020)	Fulfilled	not fulfilled	Fulfilled
Turner criteria	Not fulfilled	Fulfilled (definite)	Not fulfilled
MRI (brain, spinal cord)	No relevant findings	Cerebral atrophy, focal white matter lesions, thoracic spinal cord hypotrophy without lesions	No data available
*Neurofilaments:*			
NfL, serum	82 pg/mL	No data available	No data available
NfL, liquor	No data available	4260 pg/mL	No data available
pNfH, liquor	No data available	863 pg/mL	No data available
Chronic cough	–	+	+
sensory PNP	+ (Subclinically; axonal)	+ (Demyelinating)	+ (Axonal)
Cerebellar ataxia	–	+	–
Bilateral vestibulopathy	+ (Presbyvestibulopathy)	+	–
Autonomic dysfunction	–	+	+

## Discussion

Prompted by the discovery of pathogenic biallelic repeat expansions in *RFC1* in an ALS patient with additional sensory neuropathy and bilateral vestibular dysfunction [15], we investigated whether biallelic pathogenic repeat expansions in *RFC1* could be another relevant genetic cause for motor neuron disease such as ALS, PLS or PMA. Therefore, we systematically characterized the *RFC1* repeat array in a cohort of 107 patients with clinical diagnosis of MND (Fig. 1b, c, Supplementary Table 3). For the first time in such a cohort, repeat motifs and accurate repeat lengths of *RFC1* were determined in parallel and together with repeat expansions in *C9orf72* and *AR* by an ONT long-read sequencing method. Pathogenic biallelic repeat expansions in *RFC1* in absence of another causative genetic alteration were identified in 3% of our patients, including the patient originally identified and reported as a case report [15]. Assuming a heterozygous carrier frequency of the pathogenic ‘AAGGG’ repeat motif of up to 4% in the European population resulting in an estimated prevalence of *RFC1* spectrum disorder of up to 1/2500, individuals with a biallelic pathogenic repeat expansion were enriched by the factor 70 in this cohort, making a coincidence highly unlikely [1, 2, 31].

Interestingly, a recent study by Abramzon et al. investigated whether biallelic pathogenic repeat expansions in *RFC1* can be detected in ALS patients. When screening a cohort of 1069 patients with a clinical diagnosis of sporadic ALS, in contrast to our study, no patient with biallelic pathogenic repeat expansions in *RFC1* was found [6]. A possible explanation for these discrepant results could be the composition of the cohorts and different methods used for analyzing the *RFC1* repeat array*:* The study of Abramzon et al*.* only included ALS patients from a study registry fulfilling the El Escorial criteria, whereas in this study patients with a clinical diagnosis of MND (primarily ALS) from a real-world clinical outpatient cohort were analyzed. Another possible explanation for the identification of two patients with a clinical diagnosis of ALS in this study could be the known geographic differences in the frequencies of known ALS-associated genetic variants, even across populations with European ancestry [32]. In addition, the applied diagnostic approaches differed significantly. Abramzon et al. used an iterative PCR-based workflow without accurately determining the repeat configuration of both alleles employing ONT long-read sequencing. This might have missed patients with an *RFC1* spectrum disorder especially given the limited knowledge about the pathogenicity of individual motifs or a potential pathogenic SNV in individuals heterozygous for a pathogenic repeat expansion at that time [3, 33].

Initially, biallelic pathogenic repeat expansions in *RFC1* were identified in patients with CANVAS, a characteristic clinical triad of **c**erebellar **a**taxia, sensory **n**europathy and **v**estibular **a**reflexia [1, 2]. Meanwhile, incomplete CANVAS phenotypes and a variety of additional, atypical symptoms such as autonomic dysfunction, bradykinesia, parkinsonism or dystonia expanded the phenotype of patients carrying pathogenic repeat expansions, which led to the terminology of *RFC1* spectrum disorder [9, 34, 35]. The severity of the individual clinical presentation varies widely, and in some patients, might even fully resemble another disease phenotype, such as multiple system atrophy of the cerebellar type or parkinsonism [12, 36].

Recently, motor neuron affection has been reported as frequent additional symptom of *RFC1* spectrum disorder [13, 14]. As such, one study identified predominant affection of either the upper motor neuron in 29% or the lower motor neuron in 18% of *RFC1* patients and simultaneous affection of both, the upper and the lower motor neuron, in 16% of *RFC1* patients [13]. Furthermore, as a direct morphological correlate for clinical signs of motor neuron affection, post-mortem neuropathological examination of one patient with *RFC1* spectrum disorder showed moderate but significantly increased motor neuron loss, particularly in the anterior horn of the thoracic spinal cord and brainstem motor nuclei such as the hypoglossal nucleus. A potential mechanism of motor neuron affection is axonal swelling between the upper and lower motor neuron, which was observed in one *RFC1* patient thus leading to synaptic dysfunction [13]. This pathomechanism seems to be different to that usually observed in patients with classical ALS, which is characterized by cytoplasmic inclusions of abnormally aggregated and posttranslationally modified tar DNA binding protein (TDP-43) in neuronal cells. Postmortem neuropathological examinations of two individuals with *RFC1* spectrum disorder and motor neuron affection have shown the absence of such inclusions [35]. Thus, it seems to be the case that different pathomechanisms besides classical intraneuronal TDP-43 pathology could lead to motor neuron loss manifesting with similar clinical phenotypes in *RFC1*-pathology, but obviously with a slower progression rate as compared to classical ALS.

Besides being the origin of the distinct phenotype of CANVAS, biallelic pathogenic repeat expansions in *RFC1* might additionally induce neurodegeneration of other functional systems via yet unknown pathways. This hypothesis is in line with the recent discussion of clinical and pathological pleiotropy of neurodegenerative diseases, where similar genetic alterations can cause different phenotypes and vice versa [37]. Independent of biallelic ‘AAGGG’ repeat expansions that cause a neurodegenerative phenotype such as CANVAS with high penetrance, other *RFC1* repeat configurations could be potential genetic risk factors for a neurodegeneration such as ALS as it is known for certain intermediate and pathogenic repeat expansions such as in *ATXN1*, *ATXN2* and *HTT* [37–39]. Furthermore, biallelic pathogenic *RFC1* expansion might explain previous reports and studies with growing evidence on sensory and cerebellar involvement in ALS [40–42].

We statistically evaluated the configuration of the *RFC1* repeat array in MND patients to study its variability and a potential enrichment of distinct patterns in this cohort. However, the identification or exclusion of specific repeat configurations as risk factor for MND cannot be performed in this study. This is due to the rather small impact of polygenic risk factors on the overall disease risk, which requires larger cohorts of MND patients as well as a cohort of healthy individuals characterized by long-read sequencing to be detected.

Instead, our data highlight the intra- and interallelic heterogeneity of the *RFC1* repeat array with 6% of the alleles showing complex repeat patterns, that cannot be detected by standard PCR-based repeat analysis. Using ONT long-read sequencing for the analysis, we were able to identify what we believe to be a novel repeat motif (ACAAG)_exp_ with a size of 180 RU. Recently, it has been hypothesized that the combination of GC-content and repeat size of a certain repeat configuration determines its pathogenicity [3]. As the GC-content in’ACAAG’ is lower compared to known pathogenic repeat motifs and the detected repeat length is rather short, we postulate a non-pathogenicity of the found repeat configuration. In summary, we present repeat expansions in *RFC1* in about 3% of individuals with a clinical main phenotype of MND (i.e., two cases with ALS according to existing Gold Coast Criteria and one patient with PLS, respectively upper motor neuron dominant ALS), emphasizing that a pure or predominant motor neuron disease might be another extreme phenotype of *RFC1* spectrum disorder (see Suppl. Figure 3) and thus should be considered in the genetic diagnostic workup in patients diagnosed with MND, especially when other additional symptoms like sensory and/or autonomic neuropathy, vestibular failure, cerebellar deficits and/or a chronic cough occur. For an in-depth analysis of the heterogeneous *RFC1* locus including an accurate determination of its repeat size and motif, it is crucial to employ long-read sequencing, offering an unbiased approach to adequately capture and understand the complex genetics of *RFC1* spectrum disorder. However, further studies are needed to validate our findings and to definitely assess the role of pathogenic motifs and repeats in *RFC1* for the upper and lower motor neurons as well as to analyze a possible pathogenic role of novel repeat motifs with regard to a polygenic risk.

## Supplementary Information

Below is the link to the electronic supplementary material.

Supplementary file1 (DOCX 408 KB)

## Abbreviations

ALS: Amyotrophic lateral sclerosis
Exp: Expanded
Var: Variable
MND: Motoneuron disease
NGS: Next-generation sequencing
ONT: Oxford nanopore technologies
ROI: Region of interest
CANVAS: Cerebellar ataxia, sensory neuropathy, and vestibular areflexia syndrome
MSA: Multiple system atrophy
INDEL: Insertion and deletion
PLS: Primary lateral sclerosis
PMA: Progressive muscle atrophy
HSP: Hereditary spastic paraparesis
