# Supplementary material for: Analysis and occurrence of biallelic pathogenic repeat expansions in RFC1 in a German cohort of patients with a main clinical phenotype of motor neuron disease
Source: J Neurol. 2024 Jun 25;271(9):5804–12. doi: 10.1007/s00415-024-12519-6 (PMC11377604; doi:10.1007/s00415-024-12519-6)
Supplement: Supplementary file 1 — Supplementary file1 (DOCX 408 KB) [file 415_2024_12519_MOESM1_ESM.docx]

**Analysis and occurrence of biallelic pathogenic repeat expansions in *RFC1* in a German cohort of patients with a main clinical phenotype of motor neuron disease**

***Journal of Neurology***

**Authors:** Annalisa Schaub, Hannes Erdmann, Veronika Scholz, Manuela Timmer, Isabell Cordts, Rene Günther, Peter Reilich, Angela Abicht, Florian Schöberl

***Corresponding author:***

PD Dr. med. Florian Schöberl

Department of Neurology

Klinikum der Universität

Ludwig-Maximilians-Universität, Munich, Germany

Email: florian.schoeberl@med.uni-muenchen.de

**Supplementary Material**

**Supplementary Table 1.** CRISPR RNAs (crRNAs) to enrich repeat regions within the indicated loci.

| **Gene** | **Guide** | **Sequence 5’🡪3’** |  |
| --- | --- | --- | --- |
| *C9orf72* | C9orf72_D1 | GTTATCATCAACTTGGTACC |  |
|  | C9orf72_D2 | ATTCAAATTGAGTGAGACGG |  |
|  | C9orf72_U1 | GATGCTACAGTTACTTGATG |  |
|  | C9orf72_U2 | TAACGTAGAATAGAACCCGA |  |
| *RFC1* | RFC1_D1 | TTCGTGGAACTATCTTGGTA | |
|  | RFC1_D2 | TGATTACAACCATCAAGGAT | |
|  | RFC1_U1 | TAACTTCCAACAACCTCAAC | |
|  | RFC1_U2 | GCTCAGTCGTTTTAACCAGG | |

**Supplementary Table 2.** MND-related genes analyzed. Highlighted are genes known to be associated with ALS.

| **Gene** | | | **OMIM** | |
| --- | --- | --- | --- | --- |
| AAAS | 605378 | |  |  |
| AARS1 | 601065 | |  |  |
| ABCA1 | 600046 | |  |  |
| ABCD1 | 300371 | |  |  |
| ABHD12 | 613599 | |  |  |
| ACTA1 | 102610 | |  |  |
| ADAR | 146920 | |  |  |
| AFG3L2 | 604581 | |  |  |
| AGRN | 103320 | |  |  |
| AIFM1 | 300169 | |  |  |
| ALDH18A1 | 138250 | |  |  |
| ALG14 | 612866 | |  |  |
| ALG2 | 607905 | |  |  |
| ALS2 | 606352 | |  |  |
| AMACR | 604489 | |  |  |
| AMPD2 | 102771 | |  |  |
| ANG | 105850 | |  |  |
| ANXA11 | 602572 | |  |  |
| ANO5 | 608662 | |  |  |
| AP4B1 | 607245 | |  |  |
| AP4E1 | 607244 | |  |  |
| AP4M1 | 602296 | |  |  |
| AP4S1 | 607243 | |  |  |
| AP5Z1 | 613653 | |  |  |
| ARG1 | 608313 | |  |  |
| ARHGEF10 | 608136 | |  |  |
| ARL6IP1 | 607669 | |  |  |
| ARSA | 607574 | |  |  |
| ASAH1 | 613468 | |  |  |
| ASCC1 | 614215 | |  |  |
| ATL1 | 606439 | |  |  |
| ATL3 | 609369 | |  |  |
| ATM | 607585 | |  |  |
| ATP1A1 | 182310 | |  |  |
| ATP7A | 300011 | |  |  |
| ATP7B | 606882 | |  |  |
| B3GALNT2 | 610194 | |  |  |
| B4GALNT1 | 601873 | |  |  |
| B4GAT1 | 605517 | |  |  |
| BAG3 | 603883 | |  |  |
| BICD2 | 609797 | |  |  |
| BIN1 | 601248 | |  |  |
| BSCL2 | 606158 | |  |  |
| BTD | 609019 | |  |  |
| C19orf12 | 614297 | |  |  |
| CACNA1A | 601011 | |  |  |
| CADM3 | 609743 | |  |  |
| CAMTA1 | 611501 | |  |  |
| CAPN3 | 114240 | |  |  |
| CASQ1 | 114250 | |  |  |
| CAVIN1 | 603198 | |  |  |
| CCDC78 | 614666 | |  |  |
| CCT5 | 610150 | |  |  |
| CD59 | 107271 | |  |  |
| CFL2 | 601443 | |  |  |
| CHAT | 118490 | |  |  |
| CHCHD10 | 615903 | |  |  |
| CHKB | 612395 | |  |  |
| CHMP2B | 609512 | |  |  |
| CHRNA1 | 100690 | |  |  |
| CHRNB1 | 100710 | |  |  |
| CHRND | 100720 | |  |  |
| CHRNE | 100725 | |  |  |
| CLCF1 | 607672 | |  |  |
| CLCN2 | 600570 | |  |  |
| CLN8 | 607837 | |  |  |
| CLP1 | 608757 | |  |  |
| CNTN1 | 600016 | |  |  |
| CNTNAP1 | 602346 | |  |  |
| COA7 | 615623 | |  |  |
| COL12A1 | 120320 | |  |  |
| COL13A1 | 120350 | |  |  |
| COL6A1 | 120220 | |  |  |
| COL6A2 | 120240 | |  |  |
| COL6A3 | 120250 | |  |  |
| COLQ | 603033 | |  |  |
| COX10 | 602125 | |  |  |
| COX6A1 | 602072 | |  |  |
| CPT1C | 608846 | |  |  |
| CRLF1 | 604237 | |  |  |
| CRPPA | 614631 | |  |  |
| CTDP1 | 604927 | |  |  |
| CYP27A1 | 606530 | |  |  |
| CYP2U1 | 610670 | |  |  |
| CYP7B1 | 603711 | |  |  |
| DAG1 | 128239 | |  |  |
| DARS2 | 610956 | |  |  |
| DCAF8 | 615820 | |  |  |
| DCTN1 | 601143 | |  |  |
| DCTN2 | 607376 | |  |  |
| DDHD1 | 614603 | |  |  |
| DDHD2 | 615003 | |  |  |
| DES | 125660 | |  |  |
| DGAT2 | 606983 | |  |  |
| DHTKD1 | 614984 | |  |  |
| DMD | 300377 | |  |  |
| DNAJB2 | 604139 | |  |  |
| DNAJB5 | 611328 | |  |  |
| DNAJC12 | 606060 | |  |  |
| DNM2 | 602378 | |  |  |
| DNMT1 | 126375 | |  |  |
| DOK7 | 610285 | |  |  |
| DOLK | 610746 | |  |  |
| DPAGT1 | 191350 | |  |  |
| DPM1 | 603503 | |  |  |
| DPM2 | 603564 | |  |  |
| DPM3 | 605951 | |  |  |
| DRP2 | 300052 | |  |  |
| DST | 113810 | |  |  |
| DYNC1H1 | 600112 | |  |  |
| DYSF | 603009 | |  |  |
| EGR2 | 129010 | |  |  |
| EIF2B5 | 603945 | |  |  |
| ELP1 | 603722 | |  |  |
| EMD | 300384 | |  |  |
| ENTPD1 | 601752 | |  |  |
| EPG5 | 615068 | |  |  |
| ERLIN1 | 611604 | |  |  |
| ERLIN2 | 611605 | |  |  |
| EXOSC3 | 606489 | |  |  |
| EXOSC8 | 606019 | |  |  |
| FA2H | 611026 | |  |  |
| FBLN5 | 604580 | |  |  |
| FBXO38 | 608533 | |  |  |
| FGD4 | 611104 | |  |  |
| FHL1 | 300163 | |  |  |
| FIG4 | 609390 | |  |  |
| FKBP14 | 614505 | |  |  |
| FKRP | 606596 | |  |  |
| FKTN | 607440 | |  |  |
| FRRS1L | 604574 | |  |  |
| FUS | 137070 | |  |  |
| FXN | 606829 | |  |  |
| GAA | 606800 | |  |  |
| GAD1 | 605363 | |  |  |
| GALC | 606890 | |  |  |
| GAN | 605379 | |  |  |
| GARS1 | 600287 | |  |  |
| GBA2 | 609471 | |  |  |
| GBE1 | 607839 | |  |  |
| GBF1 | 603698 | |  |  |
| GCH1 | 600225 | |  |  |
| GDAP1 | 606598 | |  |  |
| GFPT1 | 138292 | |  |  |
| GJB1 | 304040 | |  |  |
| GJC2 | 608803 | |  |  |
| GLA | 300644 | |  |  |
| GLDC | 238300 | |  |  |
| GMPPB | 615320 | |  |  |
| GNAO1 | 139311 | |  |  |
| GNB1 | 139380 | |  |  |
| GNB4 | 610863 | |  |  |
| GPR88 | 607468 | |  |  |
| GRID2 | 602368 | |  |  |
| GSN | 137350 | |  |  |
| GYG1 | 603942 | |  |  |
| HADHA | 600890 | |  |  |
| HADHB | 143450 | |  |  |
| HARS1 | 142810 | |  |  |
| HEXA | 606869 | |  |  |
| HINT1 | 601314 | |  |  |
| HK1 | 142600 | |  |  |
| HMBS | 609806 | |  |  |
| HNRNPA1 | 164017 | |  |  |
| HNRNPA2B1 | 600124 | |  |  |
| HNRNPDL | 607137 | |  |  |
| HSPB1 | 602195 | |  |  |
| HSPB3 | 604624 | |  |  |
| HSPB8 | 608014 | |  |  |
| HSPD1 | 118190 | |  |  |
| HYCC1 | 610531 | |  |  |
| IBA57 | 615316 | |  |  |
| IFIH1 | 606951 | |  |  |
| IFRD1 | 603502 | |  |  |
| IGHMBP2 | 600502 | |  |  |
| INF2 | 610982 | |  |  |
| ITGA7 | 600536 | |  |  |
| ITPR3 | 147267 | |  |  |
| JAG1 | 601920 | |  |  |
| JPH1 | 605266 | |  |  |
| KARS1 | 601421 | |  |  |
| KBTBD13 | 613727 | |  |  |
| KDM5C | 314690 | |  |  |
| KIF1A | 601255 | |  |  |
| KIF1B | 605995 | |  |  |
| KIF1C | 603060 | |  |  |
| KIF5A | 602821 | |  |  |
| KLC4 |  | |  |  |
| KLHL13 | 300655 | |  |  |
| KLHL40 | 615340 | |  |  |
| KMT2B | 606834 | |  |  |
| KY | 605739 | |  |  |
| L1CAM | 308840 | |  |  |
| LAMA2 | 156225 | |  |  |
| LAMB2 | 150325 | |  |  |
| LAMP2 | 309060 | |  |  |
| LARGE1 | 603590 | |  |  |
| LAS1L | 300964 | |  |  |
| LIMS2 | 607908 | |  |  |
| LITAF | 603795 | |  |  |
| LMNA | 150330 | |  |  |
| LMOD3 | 616112 | |  |  |
| LRP4 | 604270 | |  |  |
| LRSAM1 | 610933 | |  |  |
| LYST | 606897 | |  |  |
| MAG | 159460 | |  |  |
| MARS1 | 156560 | |  |  |
| MARS2 | 609728 | |  |  |
| MATR3 | 164015 | |  |  |
| MCM3AP | 603294 | |  |  |
| MED25 | 610197 | |  |  |
| MEGF10 | 612453 | |  |  |
| MFN2 | 608507 | |  |  |
| MICU1 | 605084 | |  |  |
| MMACHC | 609831 | |  |  |
| MME | 120520 | |  |  |
| MORC2 | 616661 | |  |  |
| MPV17 | 137960 | |  |  |
| MPZ | 159440 | |  |  |
| MTHFR | 607093 | |  |  |
| MTM1 | 300415 | |  |  |
| MTMR2 | 603557 | |  |  |
| MTRFR | 613541 | |  |  |
| MUSK | 601296 | |  |  |
| MYH14 | 608568 | |  |  |
| MYH7 | 160760 | |  |  |
| MYO18B | 607295 | |  |  |
| MYO9A | 604875 | |  |  |
| NAGLU | 609701 | |  |  |
| NDRG1 | 605262 | |  |  |
| NDUFAF5 | 612360 | |  |  |
| NEB | 161650 | |  |  |
| NEFH | 162230 | |  |  |
| NEFL | 162280 | |  |  |
| NEK1 | 604588 | |  |  |
| NGF | 162030 | |  |  |
| NIPA1 | 608145 | |  |  |
| NKX6-2 | 605955 | |  |  |
| NT5C2 | 600417 | |  |  |
| NTRK1 | 191315 | |  |  |
| OPA3 | 606580 | |  |  |
| OPTN | 602432 | |  |  |
| ORAI1 | 610277 | |  |  |
| PANK2 | 606157 | |  |  |
| PDHA1 | 300502 | |  |  |
| PDK3 | 300906 | |  |  |
| PEX12 | 601758 | |  |  |
| PFN1 | 176610 | |  |  |
| PGAP1 | 611655 | |  |  |
| PIEZO2 | 613629 | |  |  |
| PLA2G6 | 603604 | |  |  |
| PLEC | 601282 | |  |  |
| PLEKHG5 | 611101 | |  |  |
| PLP1 | 300401 | |  |  |
| PMP2 | 170715 | |  |  |
| PMP22 | 601097 | |  |  |
| PNKP | 605610 | |  |  |
| PNPLA6 | 603197 | |  |  |
| POLG | 174763 | |  |  |
| POLR3B | 614366 | |  |  |
| POMGNT1 | 606822 | |  |  |
| POMGNT2 | 614828 | |  |  |
| POMK | 615247 | |  |  |
| POMT1 | 607423 | |  |  |
| POMT2 | 607439 | |  |  |
| PRDM12 | 616458 | |  |  |
| PREPL | 609557 | |  |  |
| PRPS1 | 311850 | |  |  |
| PRX | 605725 | |  |  |
| PTRH2 | 608625 | |  |  |
| RAB3GAP2 | 609275 | |  |  |
| RAB7A | 602298 | |  |  |
| RAPSN | 601592 | |  |  |
| RBCK1 | 610924 | |  |  |
| REEP1 | 609139 | |  |  |
| REEP2 | 609347 | |  |  |
| RETREG1 | 613114 | |  |  |
| RNASEH2B | 610326 | |  |  |
| RTN2 | 603183 | |  |  |
| RXYLT1 | 605862 | |  |  |
| RYR1 | 180901 | |  |  |
| SACS | 604490 | |  |  |
| SARS1 | 607529 | |  |  |
| SBDS | 607444 | |  |  |
| SBF1 | 603560 | |  |  |
| SBF2 | 607697 | |  |  |
| SCN10A | 604427 | |  |  |
| SCN11A | 604385 | |  |  |
| SCN4A | 603967 | |  |  |
| SCN9A | 603415 | |  |  |
| SCO2 | 604272 | |  |  |
| SELENON | 606210 | |  |  |
| SEPTIN9 | 604061 | |  |  |
| SETX | 608465 | |  |  |
| SGCA | 600119 | |  |  |
| SGCB | 600900 | |  |  |
| SGCD | 601411 | |  |  |
| SGCG | 608896 | |  |  |
| SGPL1 | 603729 | |  |  |
| SH3TC2 | 608206 | |  |  |
| SIGMAR1 | 601978 | |  |  |
| SIL1 | 608005 | |  |  |
| SLC12A6 | 604878 | |  |  |
| SLC16A2 | 300095 | |  |  |
| SLC25A1 | 190315 | |  |  |
| SLC25A21 | 607571 | |  |  |
| SLC25A26 | 611037 | |  |  |
| SLC25A46 | 610826 | |  |  |
| SLC33A1 | 603690 | |  |  |
| SLC39A14 | 608736 | |  |  |
| SLC52A2 | 607882 | |  |  |
| SLC52A3 | 613350 | |  |  |
| SLC5A7 | 608761 | |  |  |
| SMAD3 | 603109 | |  |  |
| SMN1 | 600354 | |  |  |
| SNAP25 | 600322 | |  |  |
| SOD1 | 147450 | |  |  |
| SORD | 182500 | |  |  |
| SPART | 607111 | |  |  |
| SPAST | 604277 | |  |  |
| SPG11 | 610844 | |  |  |
| SPG21 | 608181 | |  |  |
| SPG7 | 602783 | |  |  |
| SPR | 182125 | |  |  |
| SPTLC1 | 605712 | |  |  |
| SPTLC2 | 605713 | |  |  |
| SPTLC3 | 611120 | |  |  |
| SQSTM1 | 601530 | |  |  |
| STIM1 | 605921 | |  |  |
| SURF1 | 185620 | |  |  |
| SYNE1 | 608441 | |  |  |
| SYT2 | 600104 | |  |  |
| TARDBP | 605078 | |  |  |
| TBCD | 604649 | |  |  |
| TBK1 | 604834 | |  |  |
| TCAP | 604488 | |  |  |
| TDP1 | 607198 | |  |  |
| TECPR2 | 615000 | |  |  |
| TFG | 602498 | |  |  |
| TH | 191290 | |  |  |
| TIA1 | 603518 | |  |  |
| TK2 | 188250 | |  |  |
| TNNT1 | 191041 | |  |  |
| TNNT2 | 191045 | |  |  |
| TNNT3 | 600692 | |  |  |
| TNPO3 | 610032 | |  |  |
| TNXB | 600985 | |  |  |
| TOR1AIP1 | 614512 | |  |  |
| TPM2 | 190990 | |  |  |
| TPM3 | 191030 | |  |  |
| TRAPPC11 | 614138 | |  |  |
| TRIM2 | 614141 | |  |  |
| TRIM32 | 602290 | |  |  |
| TRIP4 | 604501 | |  |  |
| TRPA1 | 604775 | |  |  |
| TRPV4 | 605427 | |  |  |
| TTN | 188840 | |  |  |
| TTR | 176300 | |  |  |
| TUBA4A | 191110 | |  |  |
| TUBB3 | 602661 | |  |  |
| TUBB4A | 602662 | |  |  |
| TYMP | 131222 | |  |  |
| UBA1 | 314370 | |  |  |
| UBQLN2 | 300264 | |  |  |
| UBTF | 600673 | |  |  |
| UNC13A | 609894 | |  |  |
| USP8 | 603158 | |  |  |
| VAC14 | 604632 | |  |  |
| VAMP1 | 185880 | |  |  |
| VAPB | 605704 | |  |  |
| VCP | 601023 | |  |  |
| VMA21 | 300913 | |  |  |
| VPS13D | 608877 | |  |  |
| VPS37A | 609927 | |  |  |
| VRK1 | 602168 | |  |  |
| VWA1 | 611901 | |  |  |
| WASHC5 | 610657 | |  |  |
| WNK1 | 605232 | |  |  |
| WWOX | 605131 | |  |  |
| YARS1 | 603623 | |  |  |
| ZFYVE26 | 612012 | |  |  |
| ZFYVE27 | 610243 | |  |  |

**Supplementary Table 3. Data and results of the genetic testing of 107 patients with clinical diagnosis of MND.** All repeat lengths are given in RU. For *C9orf72* repeat lengths are only given for pathogenic repeat expansions. Patients with biallelic pathogenic repeat expansions in *RFC1* are highlighted grey. For the SNV analysis genes listed in Supplementary Table 2 were evaluated.

| **Patient ID** | **Current age** | **Gender** | ***C9orf72* repeat analysis** | ***RFC1* repeat lengths** | ***RFC1* repeat motifs** | **Main phenotype** | | **NGS panel analysis for SNVs** |
| --- | --- | --- | --- | --- | --- | --- | --- | --- |
| 1 | 66 | female | negative | 11/11 | (AAAAG)11/(AAAAG)11 | classical ALS/upper and lower motor neuron phenotype | negative | |
| 2 | 68 | female | negative | 11/11 | (AAAAG)11/(AAAAG)11 | PLS/upper motor neuron phenotype | *TARDBP* (OMIM 605078): NM_007375.4: c.881G>T p.(Gly294Val); Class 5 (PS4, PM1, PM5, PM2_SUP, PP3), heterozygous | |
| 3 | 62 | female | negative | 11/11 | (AAAAG)11/(AAAAG)11 | PLS/upper motor neuron phenotype | negative | |
| 4 | 37 | male | negative | 11/11 | (AAAAG)11/(AAAAG)11 | classical ALS/upper and lower motor neuron phenotype | negative | |
| 5 | 42 | female | negative | 11/11 | (AAAAG)11/(AAAAG)11 | classical ALS/upper and lower motor neuron phenotype | negative | |
| 6 | 85 | female | negative | 11/11 | (AAAAG)11/(AAAAG)11 | classical ALS/upper and lower motor neuron phenotype | negative | |
| 7 | 69 | male | negative | 11/11 | (AAAAG)11/(AAAAG)11 | classical ALS/upper and lower motor neuron phenotype | negative | |
| 8 | 50 | male | negative | 11/11 | (AAAAG)11/(AAAAG)11 | classical ALS/upper and lower motor neuron phenotype | negative | |
| 9 | 66 | male | negative | 11/11 | (AAAAG)11/(AAAAG)11 | classical ALS/upper and lower motor neuron phenotype | negative | |
| 10 | 62 | female | negative | 11/11 | (AAAAG)11/(AAAAG)11 | classical ALS/upper and lower motor neuron phenotype | negative | |
| 11 | 46 | male | negative | 11/11 | (AAAAG)11/(AAAAG)11 | classical ALS/upper and lower motor neuron phenotype | *FUS* (OMIM 137070): NM_004960.4: c.636C>A p.(Asp212Glu); Class 3 (PM2_SUP, PP2), heterozygous | |
| 12 | 64 | female | negative | 11/11 | (AAAAG)11/(AAAAG)11 | classical ALS/upper and lower motor neuron phenotype | negative | |
| 13 | 60 | male | negative | 11/11 | (AAAAG)11/(AAAAG)11 | PLS/upper motor neuron phenotype | negative | |
| 14 | 58 | male | negative | 11/11 | (AAAAG)11/(AAAAG)11 | classical ALS/upper and lower motor neuron phenotype | negative | |
| 15 | 63 | male | negative | 11/11 | (AAAAG)11/(AAAAG)11 | MND | negative | |
| 16 | 58 | male | positive: 6/700-2000 | 11/11 | (AAAAG)11/(AAAAG)11 | PLS/upper motor neuron phenotype | negative | |
| 17 | 50 | male | negative | 11/11 | (AAAAG)11/(AAAAG)11 | PMA/lower motor neuron phenotype | negative | |
| 18 | 32 | female | negative | 11/11 | (AAAAG)11/(AAAAG)11 | classical ALS/upper and lower motor neuron phenotype | negative | |
| 19 | 79 | male | negative | 11/11 | (AAAAG)11/(AAAAG)11 | classical ALS/upper and lower motor neuron phenotype | negative | |
| 20 | 56 | female | negative | 11/11 | (AAAAG)11/(AAAAG)11 | classical ALS/upper and lower motor neuron phenotype | negative | |
| 21 | 81 | female | negative | 11/11 | (AAAAG)11/(AAAAG)11 | classical ALS/upper and lower motor neuron phenotype | negative | |
| 22 | 60 | female | negative | 11/11 | (AAAAG)11/(AAAAG)11 | classical ALS/upper and lower motor neuron phenotype | *NEK1* (OMIM 604588): NM_001199397.3: c.271C>T p.(Arg91*); Class 4 (PVS1, PM2_SUP), heterozygous | |
| 23 | 65 | female | negative | 11/11 | (AAAAG)11/(AAAAG)11 | classical ALS/upper and lower motor neuron phenotype | negative | |
| 24 | 73 | male | negative | 11/11 | (AAAAG)11/(AAAAG)11 | classical ALS/upper and lower motor neuron phenotype | negative | |
| 25 | 51 | female | negative | 11/11 | (AAAAG)11/(AAAAG)11 | classical ALS/upper and lower motor neuron phenotype | negative | |
| 26 | 58 | female | negative | 11/107 | (AAAAG)11/(AAAAG/AAGAG)exp | classical ALS/upper and lower motor neuron phenotype | negative | |
| 27 | 58 | male | negative | 11/89 | (AAAAG)11/(AAAAG)exp | classical ALS/upper and lower motor neuron phenotype | negative | |
| 28 | 79 | female | negative | 11/101 | (AAAAG)11/(AAAAG)exp | classical ALS/upper and lower motor neuron phenotype | negative | |
| 29 | 42 | male | negative | 62/117 | (AAAAG)exp/(AAAAG)exp | classical ALS/upper and lower motor neuron phenotype | negative | |
| 30 | 55 | female | negative | 11/63 | (AAAAG)11/(AAAAG)exp | PMA/lower motor neuron phenotype | negative | |
| 31 | 54 | male | negative | 11/128 | (AAAAG)11/(AAAAG)exp | classical ALS/upper and lower motor neuron phenotype | negative | |
| 32 | 62 | male | negative | 11/115 | (AAAAG)11/(AAAAG)exp | classical ALS/upper and lower motor neuron phenotype | negative | |
| 33 | 71 | female | negative | 11/112 | (AAAAG)11/(AAAAG)exp | classical ALS/upper and lower motor neuron phenotype | negative | |
| 34 | 64 | male | negative | 11/106 | (AAAAG)11/(AAAAG)exp | classical ALS/upper and lower motor neuron phenotype | negative | |
| 35 | 55 | male | negative | 91/101 | (AAAAG)exp/(AAAAG)exp | classical ALS/upper and lower motor neuron phenotype | negative | |
| 36 | 73 | male | positive: 5/200-600 | 11/51 | (AAAAG)11/(AAAAG)exp | classical ALS/upper and lower motor neuron phenotype | negative | |
| 37 | 78 | female | negative | 11/118 | (AAAAG)11/(AAAAG)exp | PLS/upper motor neuron phenotype | negative | |
| 38 | 64 | female | negative | 11/128 | (AAAAG)11/(AAAAG)exp | classical ALS/upper and lower motor neuron phenotype | *TBK1* (OMIM 604834): NM_013254.4: c.1603G>A p.(Ala535Thr); Class 3 (PM2_SUP, BP4), heterozygous | |
| 39 | 49 | male | negative | 11/111 | (AAAAG)11/(AAAAG)exp | classical ALS/upper and lower motor neuron phenotype | negative | |
| 40 | 46 | male | negative | 90/117 | (AAAAG)exp/(AAAAG)exp | PMA/lower motor neuron phenotype | *VCP* (OMIM: 613954): NM_007126.5: c.463C>T p.(Arg155Cys); Class 5 (PS3_MOD, PS4_MOD, PM5_STR, PM2_SUP, PP1, PP3_MOD), heterozygous | |
| 41 | 59 | male | negative | 11/113 | (AAAAG)11/(AAAAG)exp | MND | negative | |
| 42 | 55 | female | negative | 11/125 | (AAAAG)11/(AAAAG)exp | classical ALS/upper and lower motor neuron phenotype | negative | |
| 43 | 76 | female | positive: 2/600-2000 | 11/98 | (AAAAG)11/(AAAAG)exp | classical ALS/upper and lower motor neuron phenotype | negative | |
| 44 | 47 | male | negative | 11/89 | (AAAAG)11/(AAAAG)exp | PLS/upper motor neuron phenotype | *SETX* (OMIM 608465): NM_015046.7: c.6652A>G p.(Met2218Val); Class 3 (PM2_SUP, BP4), heterozygous | |
| 45 | 60 | male | negative | 11/113 | (AAAAG)11/(AAAAG)exp | classical ALS/upper and lower motor neuron phenotype | negative | |
| 46 | 66 | male | positive: 2/>30 | 11/30 | (AAAAG)11/(AAAAG)exp | MND | negative | |
| 47 | 78 | male | negative | 11/94 | (AAAAG)11/(AAAAG)exp | MND | negative | |
| 48 | 73 | female | negative | 11/109 | (AAAAG)11/(AAAAG)exp | classical ALS/upper and lower motor neuron phenotype | negative | |
| 49 | 60 | male | negative | 11/109 | (AAAAG)11/(AAAAG)exp | classical ALS/upper and lower motor neuron phenotype | negative | |
| 50 | 71 | male | negative | 92/107 | (AAAAG)exp/(AAAAG)exp | PMA/lower motor neuron phenotype | negative | |
| 51 | 80 | male | negative | 92/116 | (AAAAG)exp/(AAAAG)exp | PLS/upper motor neuron phenotype | negative | |
| 52 | 56 | male | negative | 92/111 | (AAAAG)exp/(AAAAG)exp | PMA/lower motor neuron phenotype | negative | |
| 53 | 59 | female | negative | 119/138 | (AAAAG)exp/(AAAAG)exp | PLS/upper motor neuron phenotype | negative | |
| 54 | 59 | female | negative | 11/80 | (AAAAG)11/(AAAAG)exp | PLS/upper motor neuron phenotype | negative | |
| 55 | 53 | female | positive:  2/100-700 | 11/113 | (AAAAG)11/(AAAAG)exp | PLS/upper motor neuron phenotype | negative | |
| 56 | 55 | male | negative | 11/123 | (AAAAG)11/(AAAAG)exp | classical ALS/upper and lower motor neuron phenotype | negative | |
| 57 | 48 | female | negative | 111/126 | (AAAAG)exp/(AAAAG)exp | classical ALS/upper and lower motor neuron phenotype | negative | |
| 58 | 65 | female | negative | 112/120 | (AAAAG)exp/(AAAAG)exp | MND | negative | |
| 59 | 80 | male | negative | 87/95 | (AAAAG)exp/(AAAAG)exp | classical ALS/upper and lower motor neuron phenotype | negative | |
| 60 | 51 | female | negative | 11/105 | (AAAAG)11/(AAAAG)exp | classical ALS/upper and lower motor neuron phenotype | *SOD1* (OMIM 147450): NM_000454.5: c.272A>C p.(Asp91Ala); Class 3 (PS4_SUP, PP3,BS1_SUP), homozygous | |
| 61 | 76 | male | negative | 77/106 | (AAAAG)exp/(AAAAG)exp | MND | negative | |
| 62 | 64 | female | negative | 11/119 | (AAAAG)11/(AAAAG)exp | classical ALS/upper and lower motor neuron phenotype | negative | |
| 63 | 81 | male | negative | 97/125 | (AAAAG)exp/(AAAAG)exp | classical ALS/upper and lower motor neuron phenotype | negative | |
| 64 | 66 | male | negative | 11/44 | (AAAAG)11/(AAGAG)exp | PLS/upper motor neuron phenotype | negative | |
| 65 | 83 | male | negative | 11/42 | (AAAAG)11/(AAGAG)exp | classical ALS/upper and lower motor neuron phenotype | negative | |
| 66 | 69 | female | negative | 72/66 | (AAAAG)exp/(AAGAG)exp | classical ALS/upper and lower motor neuron phenotype | negative | |
| 67 | 67 | male | negative | 57/539 | (AAGAG)exp/(AAAGG)exp | PLS/upper motor neuron phenotype | negative | |
| 68 | 53 | male | negative | 101/126 | (AAAAG)exp/(AAAGG)exp | classical ALS/upper and lower motor neuron phenotype | negative | |
| 69 | 55 | male | negative | 11/476 | (AAAAG)11/(AAAGG)exp | classical ALS/upper and lower motor neuron phenotype | negative | |
| 70 | 69 | female | negative | 11/145 | (AAAAG)11/(AAAGGG)complex | classical ALS/upper and lower motor neuron phenotype | negative | |
| 71 | 41 | male | negative | 11/57 | (AAAAG)11/(AAAGGG)complex | classical ALS/upper and lower motor neuron phenotype | *SOD1* (OMIM 147450): NM_000454.5: c.43G>A p.(Val15Met); Class 5 (PS4, PM1, PM5, PM2_SUP, PP3), heterozygous | |
| 72 | 70 | female | negative | 62/66 | (AAAAG)exp/(AAAGGG)complex | classical ALS/upper and lower motor neuron phenotype | negative | |
| 73 | 61 | male | negative | 11/125 | (AAAAG)11/(AAAGGG)complex | MND | negative | |
| 74 | 54 | female | negative | 62/67 | (AAAAG)exp/(AAAGGG)complex | MND | negative | |
| 75 | 72 | male | negative | 11/86 | (AAAAG)11/(AAAGGG)complex | classical ALS/upper and lower motor neuron phenotype | negative | |
| 76 | 57 | male | negative | 11/58 | (AAAAG)11/(AAAGGG)complex | classical ALS/upper and lower motor neuron phenotype | *SOD1* (OMIM 147450): NM_000454.5: c.122T>C p.(Ile41Thr); Class 4 (PS3_MOD, PS4_MOD, PP1, PP2, PP3), heterozygous; *FIG4* (OMIM 609390): NM_014845.6: c.122T>C p.(Ile41Thr); Class 4 (PS3_MOD, PM3, PP1, PP3) | |
| 77 | 76 | male | negative | 11/130 | (AAAAG)11/(AAAGGG)complex | classical ALS/upper and lower motor neuron phenotype | negative | |
| 78 | 77 | female | negative | 11/55 | (AAAAG)11/(AAAGGG)complex | classical ALS/upper and lower motor neuron phenotype | negative | |
| 79 | 62 | male | negative | 48/88 | (AAAAG)exp/(AAAGGG)complex | classical ALS/upper and lower motor neuron phenotype | negative | |
| 80 | 66 | male | negative | 11/455 | (AAAAG)11/(AAGGG)exp | classical ALS/upper and lower motor neuron phenotype | negative | |
| 81 | 80 | female | negative | 127/405 | (AAAAG)exp/(AAGGG)exp | classical ALS/upper and lower motor neuron phenotype | negative | |
| 82 | 44 | male | positive: 2/500-2000 | 82/435 | (AAAAG)exp/(AAGGG)exp | PMA/lower motor neuron phenotype | *SOD1* (OMIM 147450): NM_000454.5: c.272A>C p.(Asp91Ala); Class 3 (PS4_SUP, PP3, BS1_SUP), heterozygous | |
| 83 | 57 | male | negative | 11/455 | (AAAAG)11/(AAGGG)exp | PMA/lower motor neuron phenotype | negative | |
| 84 | 62 | female | positive:  6/200-650 | 11/487 | (AAAAG)11/(AAGGG)exp | classical ALS/upper and lower motor neuron phenotype | negative | |
| 85 | 76 | male | negative | 41/411 | (AAAAG)exp/(AAGGG)exp | classical ALS/upper and lower motor neuron phenotype | negative | |
| 86 | 63 | male | negative | 11/487 | (AAAAG)11/(AAGGG)exp | classical ALS/upper and lower motor neuron phenotype | negative | |
| 87 | 65 | male | negative | 416/446 | (AAGGG)exp/(AAGGG)exp | classical ALS/upper and lower motor neuron phenotype | negative | |
| 88 | 53 | male | negative | 77/144 | (AAAAG)exp/(AAGGG)exp | classical ALS/upper and lower motor neuron phenotype | negative | |
| 89 | 68 | male | negative | 11/61 | (AAAAG)11/(AAAGGG)complex | classical ALS/upper and lower motor neuron phenotype | negative | |
| 90 | 77 | male | negative | 11/180 | (AAAAG)11/(ACAAG)exp | classical ALS/upper and lower motor neuron phenotype | negative | |
| 91 | 71 | female | positive: 2/100-600 | 11/43 | (AAAAG)11/(ACGGG)complex | PLS/upper motor neuron phenotype | negative | |
| 92 | 72 | female | negative | 11/11 | (AAAAG)11/(AAAAG)11 | classical ALS/upper and lower motor neuron phenotype | negative | |
| 93 | 78 | male | negative | 11/113 | (AAAAG)11/(AAAAG)exp | classical ALS/upper and lower motor neuron phenotype | negative | |
| 94 | 28 | male | negative | 11/62 | (AAAAG)11/(AAAAG)exp | PLS/upper motor neuron phenotype | negative | |
| 95 | 61 | male | negative | 11/672 | (AAAAG)11/(AAGGG)exp | classical ALS/upper and lower motor neuron phenotype | *SOD1* (OMIM 147450): NM_000454.5: c.280G>T p.(Gly94Cys); Class 5 (PM5_STR, PP1_STR, PS3_MOD, PS4_MOD, PM2_SUP, PP3), heterozygous | |
| 96 | 79 | male | negative | 11/100 | (AAAAG)11/(AAAAG)exp | classical ALS/upper and lower motor neuron phenotype | negative | |
| 97 | 75 | male | negative | 47/116 | (AAGAG)exp/(AAGGG)exp | classical ALS/upper and lower motor neuron phenotype | negative | |
| 98 | 67 | male | negative | 412/342 | (AAGGG)exp/(AAGGG)exp | PLS/upper motor neuron phenotype | negative | |
| 99 | 43 | male | negative | 560/800 | (AAGGG)exp/(AAGGG)exp | classical ALS/upper and lower motor neuron phenotype | negative | |
| 100 | 72 | male | negative | 11/108 | (AAAAG)11/(AAAAG)exp | classical ALS/upper and lower motor neuron phenotype | negative | |
| 101 | 74 | male | negative | 11/16 | (AAAAG)11/(AAAAG)exp | classical ALS/upper and lower motor neuron phenotype | negative | |
| 102 | 63 | female | negative | 11/79 | (AAAAG)11/(AAAAG)exp | classical ALS/upper and lower motor neuron phenotype | negative | |
| 103 | 69 | male | negative | 11/11 | (AAAAG)11/(AAAAG)11 | PLS/upper motor neuron phenotype | negative | |
| 104 | 68 | male | negative | 11/133 | (AAAAG)11/(AAAAG)exp | classical ALS/upper and lower motor neuron phenotype | negative | |
| 105 | 70 | male | negative | 11/80 | (AAAAG)11/(AAAAG)exp | classical ALS/upper and lower motor neuron phenotype | negative | |
| 106 | 68 | male | negative | 11/120 | (AAAAG)11/(AAAAG)exp | classical ALS/upper and lower motor neuron phenotype | negative | |
| 107 | 82 | female | negative | 11/115 | (AAAAG)11/(AAAAG)exp | classical ALS/upper and lower motor neuron phenotype | negative | |


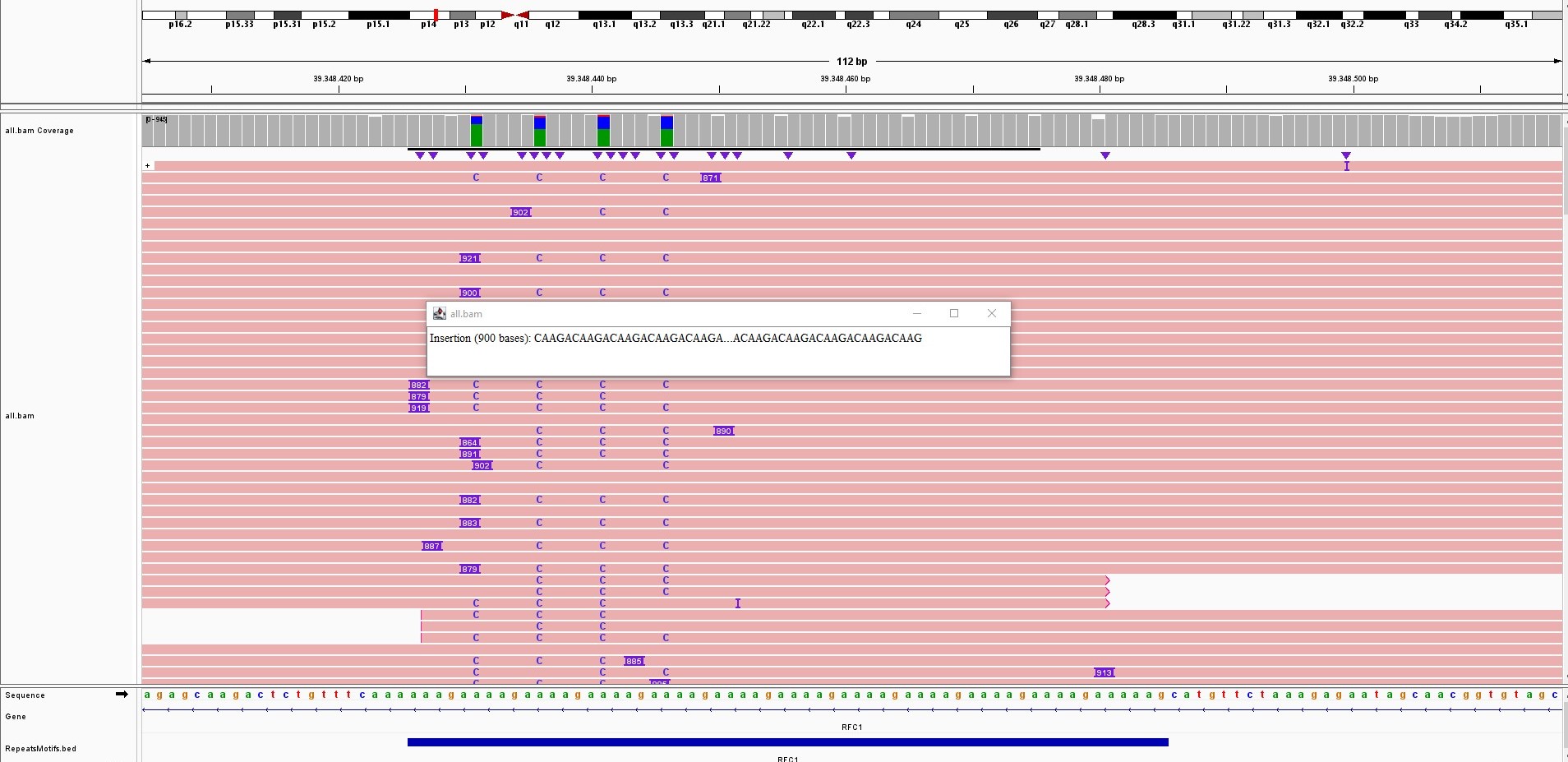


**Supplementary Figure 1. Example for the *RFC1* repeat motif ‘ACAAG’ in the IGV.** IGV (Integrative Genomics Viewer (27), version 2.16.2) presentation of the *RFC1* locus of patient #90 with insertion analysis of the repeat expansion.
